# N-back electroencephalography asymmetry and peripheral immune cell subpopulation composition

**DOI:** 10.3389/fimmu.2026.1810963

**Published:** 2026-10-06

**Authors:** Matisse Ducharme, George Nader, Vincenzo De Luca

**Affiliations:** 1 Centre for Addiction and Mental Health, Schizophrenia Department, Toronto, ON, Canada; 2 Department of Pharmacology & Toxicology, University of Toronto, Toronto, ON, Canada; 3 Department of Psychiatry, University of Toronto, Toronto, ON, Canada

**Keywords:** EEG asymmetry, electroencephalography, immune cells, immune markers, leukocytes, lymphocytes, N-back task, working memory

## Abstract

**Introduction:**

Cognitive deficits such as working memory impairment and peripheral inflammation frequently occur together in neuropsychiatric conditions, yet the relationship between them remains poorly understood. To address this gap, the present study examines these associations in healthy young adults to establish baseline relationships between peripheral immune cell subpopulations and neural activity without clinical confounds. The aim of this study is to investigate whether the relative proportions of peripheral immune cell subpopulations are associated with EEG asymmetry during a working memory task and with task performance.

**Methods:**

EEG data were recorded from 24 healthy participants during a 2-back working memory task. Relative EEG asymmetry indices were computed for four frequency bands: alpha, beta, delta, and theta. 2-back task performance was also calculated. Peripheral blood was collected for DNA extraction and genome-wide DNA methylation analysis to estimate the relative proportions of immune cell subpopulations. Estimated proportions were Center Log-Ratio-transformed. Spearman’s correlation and multiple linear regression were used to identify associations between EEG asymmetry, relative immune markers, and n-back task performance.

**Results & Discussion:**

Our analysis revealed that theta asymmetry was associated with CLR-transformed proportions of natural killer cells (p=0.035) and CD4+ T cells (p = 0.027). 2-back task target accuracy was nearly correlated with granulocytes (p = 0.082) and CD8+ T cell proportions (p=0.016); however, these associations did not remain significant after Bonferroni correction. Despite this, both immune markers significantly predicted 2-back target accuracy across three multiple linear regression models (model 1: p = 0.005, model 2: p = 0.002, model 3: p = 0.002).

## 1 Introduction

Electroencephalography (EEG) is a widely used non-invasive technique for measuring the brain’s electrical activity generated by the cortical pyramidal neurons. In cognitive neuroscience, n-back EEG recordings are commonly used to assess neural activity associated with working memory performance. EEG activity is typically analyzed across four main frequency bands including alpha waves (8–12 Hz) associated with relaxed wakefulness, beta waves (13–30 Hz) associated with active thinking and cognitive processing, delta waves (0.5–4 Hz) which predominate during deep sleep, and theta waves (4–8 Hz) associated with drowsiness and the early sleep stages (1). Notably, during working memory tasks such as the n-back task, theta activity becomes more prominent due to its involvement in cognitive processes related to episodic memory demands (2). While EEG asymmetry has been examined during resting-state recordings, its relationship to cognitive performance during a working memory task remains unexplored.

EEG asymmetry refers to the difference in electrical activity between the left and right hemispheres of the brain. Currently, literature primarily focuses on Frontal alpha asymmetry (FAA) which has been linked to affective and motivational processes, including approach-withdrawal motivation and aversion to stimuli (3), emotional responses to stimuli (4), and food-reward related inhibitory control processes (5). Additionally emerging evidence suggests that EEG asymmetry is involved in cognitive function and working memory. For example, frontal alpha and beta asymmetry have been shown to differentiate individuals with mild cognitive impairment from healthy controls and to predict cognitive performance (6). Additionally, frontal asymmetry has been shown to interact with working memory, such that asymmetry moderates the impact of working memory performance on behavioural outcomes, suggesting a functional link between hemispheric activity and executive processes (7). These studies warrant further exploration of the relationship between EEG asymmetries and cognitive function, particularly working memory. This study will be one of the first to investigate the relationship between EEG asymmetry and working memory task performance.

Furthermore, peripheral immune activity has been linked to cognitive function and working memory. Although the Central Nervous System (CNS) was once considered immunologically privileged, it has become well established that there is “cross-talk” or bidirectional communication between the peripheral immune system and the brain (8). Importantly, peripheral immune activity and central neuroinflammation are related but distinct processes, and the present study focuses specifically on peripheral immune markers. Growing evidence suggests that peripheral inflammation is associated with cognitive impairment. For example, a study by Zhuo et al. (2023) suggests a causal relationship between elevated peripheral inflammation and cognitive deterioration and structural brain changes such as reduced gray matter volume in regions involved in memory processing (9). Additionally, fluctuations in peripheral inflammation and activity have been associated with alterations in specific brain regions and functional connectivity (10). These findings suggest that peripheral immune activity or inflammation may influence cognitive and executive functions. Although it is well established that central neuroinflammation can impair brain function and contribute to cognitive deficits (11, 12), the specific peripheral immune markers that may be associated with these cognitive impairments remain poorly understood. For this reason, identifying peripheral immune cell types that may influence cognitive function could lead to the development of novel biomarkers for cognitive impairment in disorders characterized by peripheral inflammation.

In the present study, immune cell subpopulations were estimated using DNA methylation levels from peripheral blood samples. DNA methylation signatures vary across immune cell types, allowing for the inference of leukocyte subtype proportions without the need for direct immunophenotyping (13). This approach has been widely used and validated as a reliable method for quantifying immune cell composition as it is minimally invasive and a more practical technique compared to alternatives such as flow cytometry. For this reason, DNA methylation analyses provide an efficient way of assessing associations between peripheral immune activity and cognitive and electrophysiological measures.

In this study, we aim to identify potential relationships between EEG asymmetry during a working memory task (2-back), task performance, and the composition of peripheral immune cell subpopulations. By examining these three domains, we aim to determine whether specific peripheral immune cell proportions are associated with electrophysiological asymmetry and cognitive performance, particularly in the context of working memory. These relationships are examined in a healthy population to establish baseline associations in a more controlled setting, without the added complexity of confounding clinical factors. Identifying such associations in a healthy population may inform future research on peripheral immune biomarkers relevant to neuropsychiatric disorders such as schizophrenia, where working memory impairment and chronic neuroinflammation are commonly observed.

## 2 Methods

### 2.1 Participants

In this pilot study, a total of 24 healthy participants between the ages of 18 and 27 were recruited from the Centre for Addiction and Mental Health (CAMH) in Toronto. Demographic information for each participant was collected at study entry. All participants were screened and confirmed to have no psychiatric conditions using the Mini International Neuropsychiatric Interview Plus 6.0 (MINI) for DSM-IV (14). Eligibility criteria included no psychiatric diagnoses, whereas exclusion criteria for the study included the use of medications known to affect EEG recordings and working memory, a history of head trauma, and colour blindness. Additionally, the Montreal Cognitive Assessment (MOCA) and Mini-Mental State Examination (MMSE) were conducted to screen for memory impairments (15, 16). Written informed consent was obtained from all participants before study enrollment. Originally 98 individuals were enrolled in the study and had recorded EEG. Of these individuals, 24 had completed blood draws and DNA methylation analysis. The study protocol was approved by the CAMH review ethics board.

### 2.2 EEG recording and preprocessing

Continuous 2-back EEG data were recorded using NeuroScan 4.5 software and a 64-channel Quik-Cap with active scalp electrodes, placed in accordance with the International 10–20 system. A SynAmps2 amplifier was used to amplify the signals, which were then digitized at 1000 Hz using a 0.05–100 Hz band pass filter. All EEG preprocessing procedures were performed in MATLAB R2018b (17) using EEGLAB v13.6.5b (18). During preprocessing, the raw EEG data were visually screened for apparent artifacts, and any large spikes across multiple electrodes or channels exhibiting excessive noise were removed. Recordings were filtered through a zero-order bandpass from 0.5 to 40 Hz and downsampled to 250 Hz. Independent component analysis (ICA) was then applied to decompose the EEG data, allowing for the identification of non-neural artifacts, which were subsequently excluded by manual visual inspection. Common sources of artifacts included ocular movements, muscle activity, and electrical interference, such as line noise. Spherical spline interpolation was then used to interpolate previously excluded channels, and the dataset was re-referenced to the average signal of all remaining channels. Relative power spectral densities were then obtained for alpha, beta, delta, and theta frequency bands using Welch’s method with a 1-second Hanning window, 50% overlap, zero-padding, and a frequency resolution of 0.5 Hz.

### 2.3 EEG asymmetry analysis

EEG asymmetry values were calculated using the relative power spectral densities for all four frequency bands. First, hemispheric EEG asymmetry for each frequency band was calculated by summing the relative power spectral densities from the four primary midline electrodes on each hemisphere, taking the natural log of each sum, and subtracting the log-transformed left hemisphere value from the right (19).


Hemispheric Asymmetry: ln(rel(F4+C4+P4+O2))−ln(rel(F3+C3+P3+O1))

Of the 64 active electrodes, EEG asymmetry indices were calculated from eight key midline electrodes (F4, F3, C4, C3, P4, P3, O2, and O1), which are commonly used in asymmetry studies as they are less susceptible to ocular and muscle artifacts (20).

### 2.4 EEG 2-back working memory task

The 2-back task is an EEG model used to record cortical activity during a working memory task. During the 2-back task, participants were asked to monitor a randomized series of letters (stimuli) from the English alphabet appearing on a screen one at a time. Participants were asked to click a button if the current letter matched the one presented two positions earlier in the series. Each stimulus remained on the screen for 250 ms, followed by a plus sign, which gave participants 3000 ms to respond. Stimuli identical to the letter two positions back were considered “Targets”, whereas different stimuli were considered “non-targets”. Each participant monitored a total of 197 letters, including 31 targets and 166 non-targets. Participants were asked to press the “0” button when a non-target was on screen and the “1” button when a target appeared. Participants’ stimuli responses were separated into four categories: hits (target and button 1 press), false alarms (non-target and button 1 press), correct rejections (non-target and button 0 press), and misses (target and button 0 press). Performance accuracy for each participant was obtained and split into three categories: target accuracy, non-target accuracy, and d’ accuracy. Target accuracy is the fraction of correct hits, and non-target accuracy is the fraction of correct rejections, whereas d’ performance accuracy is the z-score of correctly identified targets (H) subtracted by the z-score of incorrectly identified non-targets (F), as seen below (21).


$$d^{\prime }=z(H)-z(F)$$

### 2.5 DNA sample collection

Following the EEG recording, blood samples were collected from each participant by venipuncture in vacutainers containing EDTA at CAMH. The QIAamp DNA Blood Kit (Qiagen, Hilden, Germany) was used to extract DNA from each blood sample, and the DNA samples were frozen until they were sent for DNA methylation analysis.

### 2.6 DNA methylation assay

DNA samples were diluted to 500 nanograms and sent to The Centre for Applied Genomics (TCAG) at the Hospital for Sick Children for DNA methylation analysis. DNA samples were bisulfite-converted using the EZ DNA Methylation Kit (Zymo Research, Irvine, CA, USA). Genome-wide DNA methylation (DNAm) was assessed at single-nucleotide resolution using the Infinium EPIC Methylation BeadChip (Illumina, San Diego, CA, USA), covering 850, 000 CpG loci. Poor-quality probes were defined as a detection p-value less than 0.01 and were removed using the minfi package in R studio (22, 23).

### 2.7 Immune cell subpopulation analysis

Peripheral immune cell subpopulation levels were calculated as relative proportions of the total leukocyte population based on the results from the DNA methylation analysis. DNA methylation arrays were analyzed using the Minfi package in R Studio to estimate the relative levels of the select leukocyte subpopulations (22, 23). Minfi uses a reference-based cell type deconvolution method, which compares the DNA methylation array to the IDOL reference panel dataset. Minfi then applies the Houseman regression model and decomposes the total methylation signal to estimate the proportions of CD4+ T cells, CD8+ T cells, B cells, natural killer cells, monocytes, and granulocytes. The proportions of leukocyte subpopulations were CLR-transformed to account for the compositional nature of the data, as the immune cell levels represent relative abundances that sum to a constant.

### 2.8 Statistical analysis

Data normality was assessed using the Shapiro-Wilk test in IBM SPSS Statistics 27 (24), which identified that n-back accuracy data and immune cell subpopulation proportions were not normally distributed. Therefore, Spearman’s rho was used to identify any associations between EEG asymmetry indices, CLR-transformed immune cell subpopulation proportions, and 2-back target accuracy. P-values < 0.05 were considered nominally significant; however, a Bonferroni correction was applied to establish the final significance threshold. Additionally, a power analysis using G*Power (Version 3.1.9.7) indicated that the study could achieve 80% power to detect correlations of approximately r = 0.52, using a sample size of 24 and an α = 0.05. Associations of nominal significance were then included in various multiple linear regression models, adjusting for age, in which a p-value of <0.05 was considered significant. Multiple linear regressions were performed in SPSS, in which standardized regression coefficients (β) were computed by internally standardizing variables (z-scores) prior to model estimation.

## 3 Results

### 3.1 Demographics

Demographic information for the 24 healthy participants is available in **Table 1**. The mean age of participants was 20.92 ± 2.89 years and the cohort was made up of 11 males (46%) and 13 females (54%). The ethnicity of each individual was based on the ethnicity of their four grand-parents, resulting in a mostly mixed background with the majority being European/Caucasian (34.4%). Additionally, **Table 2** shows the average relative distribution of leukocyte subtypes.

**Table 1:** Clinical demographic information for participants (n=24).

Age (Years; mean ± SD)	20.92 ± 2.89
Sex [n (%)]	
Male	11 (46%)
Female	13 (54%)
Other	0 (0%)
Ethnicity [n (%)]	
African Descent/African American	3 (12.5%)
European/Caucasian	8.25 (34.4%)
Asian	4.75 (19.8%)
Hispanic/Latino	2.5 (10.4%)
East Indian Caucasian	5.25 (21.9%)
Pacific Islander	0.25 (1%)

**Table 2:** Average distribution of relative peripheral leukocyte subpopulations.

Leukocyte subpopulation	Average ± SD
CD8^+^T cells	0.102 ± 0.044
CD4^+^T cells	0.170 ± 0.046
Natural Killer cells	0.047 ± 0.025
B Cells	0.072 ± 0.019
Monocytes	0.057 ± 0.017
Granulocytes	0.599 ± 0.091

### 3.2 Correlation analysis

A correlation analysis was completed to investigate any potential associations between hemispheric asymmetry in each frequency band and the CLR-transformed immune cell proportions. Due to the lack of normality of the immune cell data, Spearman’s rho was used to assess the associations. Two significant correlations were identified with hemispheric theta asymmetry: a negative correlation with CLR-transformed CD4+T cell levels (r = -0.45, p = 0.027) and a positive correlation with CLR-transformed natural killer cell levels (r = 0.431, p = 0.035). As well, a positive correlation was identified between delta asymmetry and CLR-transformed natural killer cells (r = 0.409, p = 0.047). However, these associations did not remain significant when applying a Bonferroni-corrected p-value. Correlation analyses for hemispheric asymmetry and CLR-transformed immune cell subpopulation proportions are displayed in **Figure 1A**.

**Figure 1:**
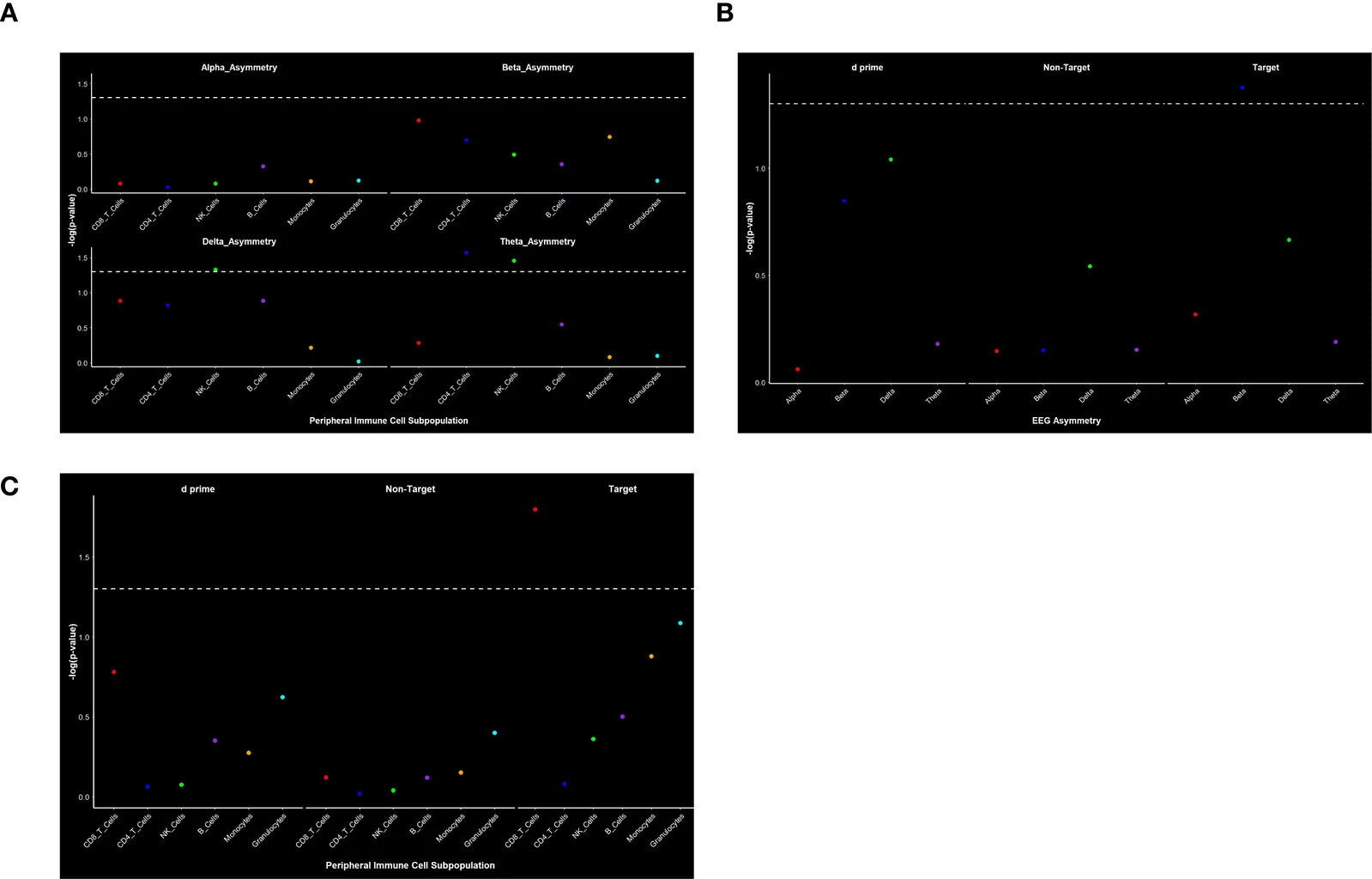
Manhattan plots showing correlation analysis of A) Hemispheric asymmetry with CLR-transformed lymphocyte subtype proportions B) Hemispheric asymmetry and 2-back task performance accuracy. C) CLR-transformed lymphocyte subtypes proportions and 2-back task performance accuracy. The dotted line represents p = 0.05.

A correlation analysis was also used to explore the relationship between 2-back target, non-target, and d’ accuracy and hemispheric asymmetry. We identified a positive correlation between the 2-back target accuracy and Beta asymmetry (r = 0.419, p = 0.042). The results are shown in **Figure 1B**.

Similarly, associations between 2-back task performance and CLR-transformed immune cell proportions were also explored in a correlation analysis. This analysis showed a significant positive association between 2-back target accuracy and CLR-transformed CD8+T cell levels (r = 0.486, p = 0.016). Additionally, a negative correlation between target accuracy and CLR-transformed granulocyte levels approached significance (r = -0.362, p = 0.082). **Figure 1C** shows the correlation analysis between 2-back task accuracy and CLR-transformed peripheral immune cell subpopulation levels.

### 3.3 Hemispheric theta asymmetry

To better understand the relationship between hemispheric theta asymmetry, CD4+T cells, and natural killer cells, three multiple linear regression models were conducted. The first model included CLR-transformed levels of both CD4+T and natural killer cells as predictors, with age included as a covariate. However, due to the high collinearity between CD4+T cells and NK cells (VIF >5 in Model 1), the predictors were separated into two additional models to assess their individual relationships with theta asymmetry. **Table 3** presents the multiple linear regression coefficients for each predictor across the three models, including unstandardized (B) and standardized (Beta) coefficients, standard errors (SE), p-values, and variance inflation factors (VIF). As well, **Table 4** summarizes the overall model fit statistics for each regression.

**Table 3:** Multiple linear regression models predicting hemispheric theta asymmetry.

Predictor	B	SE	Beta	P-value	VIF	Model
CD4^+^T	0.036	0.087	0.203	0.684	5.137	1
NK	0.047	0.055	0.430	0.403	5.402	1
Age	-0.003	0.008	-0.088	0.702	1.102	1
CD4^+^T	-0.031	0.039	-0.173	0.435	1.025	2
Age	-0.001	0.007	-0.037	0.868	1.025	2
NK	0.026	0.024	0.244	0.279	1.071	3
Age	-0.002	0.007	-0.072	0.746	1.071	3

**Table 4:** Summary statistics for multiple linear regression models predicting hemispheric theta asymmetry.

Model	R^2^	Adjusted R^2^	F	df	P-value
1	0.064	-0.077	0.453	3, 20	0.718
2	0.029	-0.063	0.318	2, 21	0.731
3	0.056	-0.034	0.619	2, 21	0.548

Model 1 examined whether CLR-transformed CD4^+^ T cell levels, natural killer cell levels, and age significantly predicted hemispheric theta asymmetry using multiple linear regression:

Theta Asymmetry = β_0_ + β_1_(CD4^+^ T) + β_2_(NK) + β_3_(Age) + ϵ

Model 2 included CD4^+^ T cells and age:

Theta Asymmetry = β_0_ + β_1_(CD4^+^ T) + β_2_(Age) + ϵ

Model 3 included natural killer cells and age only:

Theta Asymmetry = β_0_ + β_1_(NK) + β_2_(Age) + ϵ

All three multiple linear regression models assessed were not significant for accurately predicting theta asymmetry as shown in **Table 4**.

### 3.4 2-back performance accuracy

Additionally, three multiple linear regression models were conducted to explore the relationship between 2-back target accuracy, CLR-transformed CD8+ T cells, and granulocytes. The first model included both CD8+ T cells and granulocytes as predictors and age as a covariate. However, due to the high collinearity between CD8+ T cells and granulocytes (VIF >6 in Model 1), the predictors were then separated into two additional models to assess the individual relationships with 2-back target accuracy. **Table 5** presents the multiple linear regression coefficients for each predictor across the three models. As well, **Table 6** summarizes the overall model fit statistics for each regression.

**Table 5:** Multiple linear regression models predicting 2-back target accuracy.

Predictor	B	SE	Beta	P-value	VIF	Model
CD8^+^ T	0.073	0.078	0.400	0.361	6.804	1
Granulocytes	-0.058	0.133	-0.404	0.666	6.745	1
Age	0.016	0.011	0.157	0.147	1.051	1
Granulocytes	-0.172	0.052	-0.553	0.003*	1.042	2
Age	0.017	0.010	0.268	0.123	1.042	2
CD8^+^ T	0.104	0.030	0.573	0.002*	1.051	3
Age	0.016	0.010	0.253	0.140	1.051	3

**Table 6:** Summary statistics for multiple linear regression models predicting 2-back target accuracy. *: p<0.01

Model	R^2^	Adjusted R^2^	F	df	P-value
1	0.461	0.380	5.709	3, 20	0.005*
2	0.438	0.384	8.175	2, 21	0.002*
3	0.456	0.404	8.805	2, 21	0.002*

Model 1 examined whether CLR-transformed CD8^+^ T cell levels, granulocyte levels, and age significantly predicted N-back target accuracy using multiple linear regression:

Target Accuracy = β_0_ + β_1_(CD8^+^ T) + β_2_(Granulocyte) + β_3_(Age) + ϵ

Model 2 included CD8^+^ T cells and age:

Target Accuracy = β_0_ + β_1_(CD8^+^ T) + β_2_(Age) + ϵ

Model 3 included granulocytes cells and age only:

Target Accuracy = β_0_ + β_1_(Granulocytes) + β_2_(Age) + ϵ

All three multiple linear regression models were statistically significant (p = 0.005 in Model 1, p = 0.002 in Model 2, and p = 0.002 in Model 3). The greatest statistical significance was seen in Model 2, which included granulocytes as the predictor and age as a covariate (**Table 6**). In this model, the coefficient for granulocytes was statistically significant with a p-value of 0.003. In model 3, CD8+ T cells also had a significant coefficient with a p-value of 0.002 (**Table 5**).

## 4 Discussion

In this study, we identified two associations with hemispheric theta asymmetry, including CLR-transformed levels of natural killer cells and CD4^+^ T cells. After investigating further, we determined that the transformed proportions of natural killer cells were positively correlated with theta asymmetry. In contrast, the transformed proportions of CD4^+^ T cells were negatively associated with theta asymmetry. However, when assessing whether the CLR-transformed levels of natural killer and CD4^+^ T cells were predictive of theta asymmetry using multiple linear regression models, the results were not statistically significant. Studies have shown that during task-related EEG measures such as n-back tasks, increased theta activity is associated with better cognitive task performance. That is reasonable as theta waves are associated with various cognitive functions such as attention, memory, and emotional processing (25). However, its relationship with immune markers such as natural killer and CD4^+^ T cells has never been studied, making potential interpretations of the identified associations difficult.

However, it is important to note that these findings were observed in a healthy population without evidence of peripheral inflammation or immune dysregulation. As such, the identified associations likely reflect a subtle variation in baseline immune system composition rather than pathological immune activation. These associations suggest that even under normal physiological conditions, individual differences in peripheral immune cell distributions may be associated with variability in cognitive performance and neural activity. These relationships may be mediated through neuroimmune signaling pathways such as cytokine communication or indirect modulation of neural function. However, future studies in healthy adults should be conducted to better understand the underlying pathophysiology of these relationships. Therefore, the present findings may reflect typical immune-brain interactions that contribute to variability in brain electrical activity and cognitive function.

Additionally, in this study, we identified one significant association with 2-back target accuracy and CLR-transformed levels of CD8^+^ T cells, and one association nearing significance between 2-back target accuracy and granulocytes. To further investigate these relationships, we conducted three multiple linear regression models to assess whether transformed granulocytes and CD8^+^ T cell levels are predictive of 2-back target accuracy, while controlling for age. All three models were found to be statistically significant, with granulocyte levels being the most predictive of EEG 2-back target accuracy. CLR-transformed proportions of granulocyte levels were negatively associated with target accuracy, indicating that a higher relative abundance of granulocytes was linked to decreased task performance. Whereas, transformed CD8^+^ T cell proportions were positively associated with target accuracy, meaning increases in the relative proportions of CD8^+^ T cells are predictive of better target accuracy.

Granulocytes are a type of effector cell involved in the innate immune response. They are predominantly made up of neutrophils, as well as eosinophils, basophils, and mast cells (26). Neutrophils are a key component of the peripheral immune system, the first responders to pathogens and tissue damage, and the most abundant immune cell in humans (27, 28). Due to the “cross-talk” between the peripheral immune system and the brain, the relationship between various peripheral immune cells and cognition has become of great interest. In a study by Fa et al., it was determined that a high absolute neutrophil count was associated with lower performance in episodic memory tests, suggesting that neutrophils may play a role in cognitive aging and memory decline (29). The above mentioned study also found that higher levels of serum cytokines, such as IL-6 and eotaxin-3, are associated with poor memory function and lower performance in memory function (29). Since IL-6 and eotaxin-3 play a role in the regulation and recruitment of neutrophils, they may play a role in mediating the relationship between elevated absolute neutrophil count and cognitive deficits.

Other studies have also identified links between elevated neutrophil counts and cognitive deficits such as memory impairment. In transgenic mice models of Alzheimer’s disease, it was found that neutrophils were able to infiltrate mice brains and release neutrophil extracellular traps near the amyloid-β plaques associated with the disease (30). The release of neutrophils in mouse brains resulted in increased oxidative stress, tissue damage, and chronic neuroinflammation. However, when these neutrophils were depleted, the mouse models showed improved memory and cognitive performance (28, 30). The discovery that neutrophils may play a role in the cognitive decline seen in diseases such as Alzheimer’s makes it clear that elevated neutrophil levels may play an important role in the impairment of working memory. It is possible that a higher neutrophil ratio is indicative of increased neuroinflammation, likely mediated by the release of cytokines. Therefore, the relationship between granulocytes and 2-back target accuracy may be mediated by neutrophil levels and its resulting inflammation.

Understanding the links between the immune system and cognition, particularly working memory, is crucial for understanding certain disorders. For example, patients with schizophrenia often struggle with working memory impairments, however, the mechanisms of these impairments are not fully understood (31, 32). Studies have also shown that elevated neutrophil counts and neutrophil-to-lymphocyte ratios are often seen in patients with schizophrenia (33). Although the brain was once thought to be “immune-privileged “ it has become increasingly clear that during inflammation, immune cells such as neutrophils are capable of passing the blood-brain barrier (BBB) and interacting with neural tissue. However, neutrophil activity in the brain often causes neuroinflammation due to the release of cytokines, chemokines, and reactive oxygen species (34). In schizophrenia, particularly, the BBB has been shown to have increased permeability, which may in part be caused by increased neutrophil activity (34). Due to the ability of neutrophils to influence synaptic strength and plasticity, it has been hypothesized that neutrophil activity may play a role in the cognitive and memory deficits seen in schizophrenia. Therefore, the excess neuroinflammation, neutrophil activity, and pro-inflammatory cytokine release seen in schizophrenia may play a key role in the impairments of cognition and working memory. However, the present study was conducted in a population of healthy adults, where BBB impairment and peripheral inflammation are unlikely. As such, these mechanisms are unlikely to directly account for the observed associations. The identified relationships may instead reflect indirect forms of neuroimmune communication under normal physiological conditions. Compared to neutrophils, the role of CD8^+^ T cells in cognition and working memory is much less clear. CD8^+^ T cells play a multitude of roles in the adaptive immune system due to their ability to differentiate into a variety of CD8^+^ cell types. For example, while most CD8^+^ T cells act as cytotoxic T lymphocytes that recognize and remove infected cells, a small portion can differentiate into regulatory T cells (CD8^+^ Tregs) able to regulate and suppress the immune system (35). The majority of studies investigating the relationship between CD8^+^ T cells and cognition or memory have been conducted within the context of Alzheimer’s disease models, however, these studies are often contradictory. In some mouse models of Alzheimer’s disease, the recruitment and migration of CD8^+^ T cells to the β-amyloid plaques resulted in decreased inflammation and a reduction of β-amyloid plaques as well as improvement in cognitive decline (36). In this model the CD8^+^ T cells acted as protective CD8^+^ Tregs to reduce inflammation. However, in other mouse models of Alzheimer’s disease, CD8^+^ T cell recruitment was identified to worsen inflammation and contribute to the neurodegeneration seen in Alzheimer’s (12). Due to the conflicting roles of CD8^+^ T cells, it is unclear how these cells may be predictive of 2-back target accuracy and working memory performance.

This study has various limiting factors. For one, the sample size of this study is small and not representative of the general population, as the participants were similar in age and education status. A larger and more diverse sample size may increase statistical power, potentially resulting in associations that remain significant after using the Bonferroni correction. In the future, this study should be replicated using a larger sample size for greater power. As well, the study is limited by the use of only 8 main electrodes for the EEG asymmetry analysis. The use of all 64 active electrodes could improve the spatial resolution of the EEG data however, only 8 electrodes were used to avoid excessive artifacts and prioritize high-quality data. Future studies should work on replicating this study using all 64 active electrodes to enhance spatial resolution. Additionally, the peripheral immune cell subpopulation proportions were estimated from the DNA methylation analysis, which could introduce error since it does not measure the immune cells directly. To avoid this limitation, future studies could use methods such as flow cytometry to get an accurate measurement of the immune cell levels. It would also be of great interest to conduct a similar study in individuals with disorders such as schizophrenia and Alzheimer’s disease, as this study may be beneficial in the search of potential novel biomarkers in disorders associated with working memory impairment as well as neuroinflammation. Finally, it is important to note that this study did not assess the effects of potentially confounding variables such as participant infections, allergies, life stressors, and current living environments. Although all participants were confirmed for the absence of any current diseases, these factors can still impact immune activity and therefore impact the results. Future studies should assess the potential impacts of these confounding variables.

In conclusion, this small exploratory study assessing the relationship between EEG asymmetry during a working memory task, task performance and relative peripheral immune cell subtype levels, provides a new way of understanding how the immune system may impact cognitive function and working memory. This study provides the groundwork for future studies analyzing how immune markers may impact cognitive dysfunction and memory impairment in relevant neuropsychiatric disorders.

## Data Availability

The raw data supporting the conclusions of this article will be made available by the authors, without undue reservation.
